# Retinoic acid regulates erythropoietin production cooperatively with hypoxia-inducible factors in human iPSC-derived erythropoietin-producing cells

**DOI:** 10.1038/s41598-021-83431-6

**Published:** 2021-02-16

**Authors:** Naoko Katagiri, Hirofumi Hitomi, Shin-Ichi Mae, Maki Kotaka, Li Lei, Takuya Yamamoto, Akira Nishiyama, Kenji Osafune

**Affiliations:** 1 Department of Cell Growth and Differentiation, Center for iPS Cell Research and Application (CiRA), Kyoto University, Kyoto, 606-8507 Japan; 2 Department of iPS Stem Cell Regenerative Medicine, Kansai Medical University, Osaka, 573-1010 Japan; 3 Department of Pharmacology, Faculty of Medicine, Kagawa University, Kagawa, 761-0793 Japan; 4 Department of Life Science Frontiers, CiRA, Kyoto University, Kyoto, 606-8507 Japan; 5 Institute for the Advanced Study of Human Biology (WPI-ASHBi), Kyoto University, Kyoto, 606-8501 Japan; 6 AMED-CREST, AMED 1-7-1 Otemachi, Chiyodaku, Tokyo, 100-0004 Japan; 7 Medical-Risk Avoidance Based on iPS Cells Team, RIKEN Center for Advanced Intelligence Project (AIP), Kyoto, 606-8507 Japan

**Keywords:** Regeneration, Stem cells

## Abstract

Erythropoietin (EPO) is a crucial hormone for erythropoiesis and produced by adult kidneys. Insufficient EPO production in chronic kidney disease (CKD) can cause renal anemia. Although hypoxia-inducible factors (HIFs) are known as a main regulator, the mechanisms of EPO production have not been fully elucidated. In this study, we aimed to examine the roles of retinoic acid (RA) in EPO production using EPO-producing cells derived from human induced pluripotent stem cells (hiPSC-EPO cells) that we previously established. RA augmented EPO production by hiPSC-EPO cells under hypoxia or by treatment with prolyl hydroxylase domain-containing protein (PHD) inhibitors that upregulate HIF signals. Combination treatment with RA and a PHD inhibitor improved renal anemia in vitamin A-depleted CKD model mice. Our findings using hiPSC-EPO cells and CKD model mice may contribute to clarifying the EPO production mechanism and developing efficient therapies for renal anemia.

## Introduction

Erythropoietin (EPO) is a potent regulatory hormone for erythropoiesis and mainly produced by adult kidneys^1^. Renal anemia resulting from decreased EPO production in patients with chronic kidney disease (CKD) has been treated with recombinant human EPO (rhEPO) agents^2^. However, intermittent treatments with rhEPO increase the risk of cardiovascular diseases^3^, thus more physiological therapies are required.

EPO production is regulated by hypoxic signals to control erythropoiesis. Although the hypoxia-inducible factor (HIF)-prolyl hydroxylase domain-containing protein (PHD) pathway is known as a main regulator, the detailed mechanism of EPO production, especially in humans, has not been fully elucidated^4^. Recently, the use of PHD inhibitors, which stabilize HIFs, has been started as therapeutic agents for renal anemia. However, there have been concerns about the risks of side effects, including the development of malignant tumors and retinopathy, because HIFs regulate numerous genes related to tumorigenesis, angiogenesis, glycolysis and cell proliferation^5,6^.

In vitro drug screenings and mechanistic analyses of EPO production via the HIF pathway are difficult to perform using human EPO-producing cells isolated from kidneys because of the limited access and difficulty in maintaining the cells in vitro. Since EPO is also produced by the liver during fetal and early neonatal periods and even by adult liver in the case of severe anemia, the liver is a potential target of new therapeutic strategies for renal anemia besides the kidneys^1,7^. We previously developed human induced pluripotent stem cell (hiPSC)-derived EPO-producing cells (hiPSC-EPO cells) by modifying previously reported hepatic differentiation protocols^8^. EPO production and secretion were observed in hiPSC-EPO cells in an oxygen-dependent manner and increased by PHD inhibitors in vitro. In addition, cell therapy using hiPSC-EPO cells showed long-term therapeutic effects for renal anemia in a CKD mouse model. Hence, hiPSC-EPO cells can be a beneficial tool to elucidate the mechanisms of EPO production in vitro and in vivo.

Retinoic acid (RA) is a metabolite of vitamin A and involved in broad physiological processes including embryonic development, immunity, cellular differentiation and proliferation, mainly by regulating gene expressions^9–11^. RA activates two classes of nuclear receptors, RA receptors (RARs) and retinoid X receptors (RXRs), and each receptor has three subtypes, RARα, β and γ and RXRα, β and γ, respectively. RARs form heterodimers with RXRs^12,13^ that bind to specific DNA regions termed RA response elements (RAREs), which control gene transcription. RA is essential for the hepatic production of EPO in early developmental stages^14,15^. Importantly, almost no notable side effects on the clinical use of RA have been reported except for teratogenicity^16^. Several studies also reported that RA inhibits neovascularization by inhibiting vascular endothelial growth factor (VEGF) expression in tumor cells or an animal model of retinopathy of prematurity^17,18^. For these reasons, RA could be a safe and low-cost therapeutic agent for renal anemia. However, the molecular mechanisms of EPO production regulated by RA signals alone or with the HIF-PHD pathway remain to be elucidated. Especially, the effects of combination treatments with RA and PHD inhibitors have not been investigated in any experimental animal model.

In the present study, we aimed to clarify the mechanisms of EPO production via RA signals and the HIF-PHD pathway using hiPSC-EPO cells and the effects of combination treatment with RA and PHD inhibitors in a mouse model of renal anemia. We confirmed that RA increases the EPO expression and secretion by hiPSC-EPO cells and additively augments the EPO production induced by PHD inhibitors. We also examined the epigenome changes to clarify the mechanisms by which RA and HIF signals regulate the EPO production. Furthermore, we demonstrated that combination treatment with RA and a PHD inhibitor increases the EPO production in ex vivo tissue cultures of adult mouse kidneys and that the PHD inhibitor improves renal anemia in the presence of RA in CKD mouse models.

## Results

### RA signals increase EPO production additively with hypoxic signals in hiPSC-EPO cells

**Figure 1:**
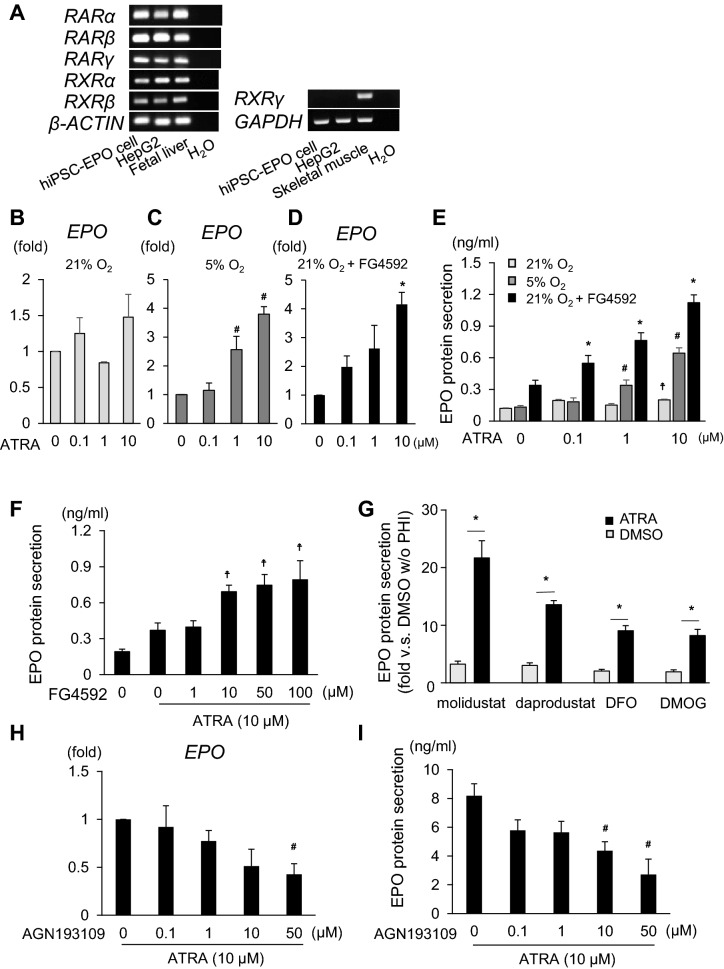
Effects of ATRA and hypoxic signals on EPO production by hiPSC-EPO cells. (**A**) Semiquantitative RT-PCR analysis of the mRNA expression of *RARs* and *RXRs* by hiPSC-EPO cells. HepG2 cells, human fetal liver tissues and human skeletal muscle tissues were used as positive controls. Cropped gels are displayed. (**B**–**E**) Effects of ATRA treatment on EPO mRNA expression (**B**–**D**) and protein secretion (**E**) by hiPSC-EPO cells under normoxia (21% oxygen; **B**,**E** light gray), hypoxia (5% oxygen; **C**,**E**, dark gray) and normoxic conditions combined with PHD inhibitor treatment (10 μM FG4592; **D**,**E**, black), as analyzed by qRT-PCR and ELISA, respectively. Note that the analyses in (**B**–**D**) were performed independently. (**F**) Concentration-dependent effects of FG4592 on EPO protein secretion by hiPSC-EPO cells treated with 10 μM ATRA under normoxic conditions. (**G**) Effects of ATRA combined with several PHD inhibitors (100 μM molidustat, daprodustat and DFO, and 1 mM DMOG) on EPO protein secretion by hiPSC-EPO cells under normoxic conditions. (**H**,**I**) Effects of adding various concentrations of an RARα antagonist, AGN193109, to the ATRA treatment on EPO mRNA expression (**H**) and protein secretion (**I**) by hiPSC-EPO cells under hypoxic conditions. The data from four (n = 4 for **B**—**E**, **H** and n = 6 for **I**) or three independent experiments (n = 3 for **F**, **G**) are represented as the means ± SEM in (**B**–**I**). Statistical analysis was performed using one-way ANOVA with Dunnett’s multiple comparison test in (**B**–**F**,**H**,**I**) and Student’s t test in (**G**). ^#^p < 0.05 versus the samples treated with DMSO under hypoxic conditions in (**C**,**E**) and those treated with ATRA but without AGN193109 under hypoxic conditions in (**H**,**I**). *p < 0.05 versus the samples treated with PHD inhibitors but without ATRA under normoxic conditions in (**D**,**E**,**G**). ^☨^p < 0.05 versus the samples treated with DMSO under normoxic conditions in (**E**,**F**).

Previous studies reported that EPO production depends on RA signals in the early developmental stages of embryonic liver^14,19^. Therefore, we examined the roles of RA as a regulator of EPO production in hiPSC-EPO cells that were generated by modifying previously reported hepatic differentiation protocols^8^. First, we examined the expression of RAR and RXR subunits in hiPSC-EPO cells. Semiquantitative RT-PCR analyses revealed that hiPSC-EPO cells express the mRNAs of *RARα, β* and *γ* and *RXRα* and *β*, but not *RXRγ* (Fig. 1A, supplementary Fig. S3).

To evaluate how RA signals work in EPO production, we investigated the effects of two different retinoids, all-trans retinoic acid (ATRA) and bexarotene. ATRA is the major RA, is present in high abundance in the body compared to its isomer, 9-cis RA, and acts by binding to RARs^13^. Bexarotene is a synthetic analog that selectively binds to RXR^20^. Under normoxic conditions (21% oxygen), only more than 10 μM ATRA slightly but significantly increased EPO protein secretion (Fig. 1B,E, supplementary Fig. S1A), and 0.1 and 1 μM bexarotene weakly increased *EPO* mRNA expression (supplementary Fig. S1B,E). By contrast, both ATRA and bexarotene at 1 and 10 μM significantly increased EPO mRNA expression and protein secretion under hypoxic conditions (5% oxygen; Fig. 1C,E, supplementary Fig. S1C,E). Especially, ATRA increased EPO mRNA expression and protein secretion in a dose-dependent manner. In order to more accurately examine the interaction effects between RA signals and the HIF-PHD pathway, we evaluated the effects of combination treatment with RA and PHD inhibitors. The results showed that ATRA additively increased EPO mRNA expression and dose-dependently increased protein secretion with 10 μM FG4592 under normoxic conditions (Fig. 1D,E). We also examined the dosage effects of FG4592 under treatment with 10 μM ATRA, finding more than 10 μM FG4592 additively increased EPO protein secretion by hiPSC-EPO cells (Fig. 1F). ATRA also additively increased the EPO protein secretion with other PHD inhibitors, such as molidustat^21^, daprodustat^22^, an iron chelator, deferoxamine (DFO)^23^, and a 2-oxoglutarate analog, dimethyloxalylglycine (DMOG)^23^ (Fig. 1G). On the other hand, combination treatment with bexarotene and FG4592 did not show an additive effect on EPO production under normoxic conditions except for EPO protein secretion at 1 μM bexarotene (supplementary Fig. S1D,E). To prove the individual effects of RAR, an antagonist was examined. We confirmed that a pan-RAR antagonist, AGN193109, attenuated both EPO mRNA expression and protein secretion by hiPSC-EPO cells treated with ATRA under hypoxic conditions (Fig. 1H,I). These results suggest that RA signals, especially those through RARs, are crucial for EPO production regulated by the HIF-PHD pathway in hiPSC-EPO cells.

### RA does not regulate EPO production through the proliferation or differentiation of hiPSC-EPO cells or the expression of HIFs and their regulators

**Figure 2:**
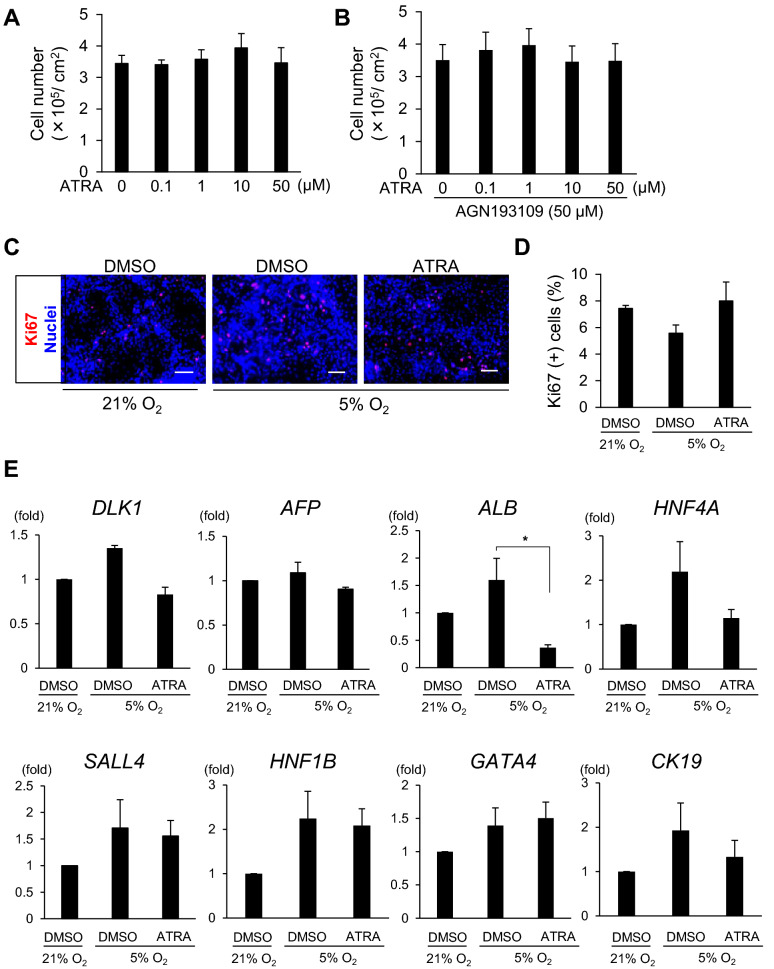
Effects of RA on the proliferation and differentiation of hiPSC-EPO cells. (**A**,**B**) The numbers of hiPSC-EPO cells after treatment with ATRA alone (**A**) or combined with AGN193109 (**B**) under hypoxic conditions (5% oxygen). (**C**) Immunostaining analysis of hiPSC-EPO cells for a proliferation marker, Ki67. Scale bars 100 μm. (**D**) Percentage of Ki67(+) cells in (**C**). (**E**) qRT-PCR analysis of the expression of the hepatic lineage markers, *DLK1*, *AFP*, *ALB*, *HNF4A, SALL4*, *HNF1B*, *GATA4* and *CK19*, by hiPSC-EPO cells treated with DMSO under normoxic (21% oxygen) or hypoxic conditions or with ATRA under hypoxic conditions. The data from three independent experiments (n = 4 for **A**,**B** and n = 3 for **D**,**E**) are represented as the means ± SEM in (**A**,**B**,**D**,**E**). Statistical analysis was performed using one-way ANOVA with Bonferroni’s test in (**A**,**B**,**D**,**E**). *p < 0.05 versus the samples treated with DMSO under hypoxic conditions.

In an attempt to clarify the regulatory mechanisms of EPO production by RA and hypoxic signals in hiPSC-EPO cells, we first evaluated the effects of ATRA on the proliferation and differentiation status of hiPSC-EPO cells. To evaluate the possibility of cell proliferation via RA signals, we counted the numbers of hiPSC-EPO cells treated with ATRA alone or with ATRA and AGN193109 under hypoxic conditions but found no significant differences at various concentrations (Fig. 2A,B). The frequency of cells positively stained with a cell proliferation marker, Ki67, was comparable between cells treated with dimethyl sulfoxide (DMSO) under normoxic and hypoxic conditions and between those treated with DMSO or ATRA under hypoxic conditions (Fig. 2C,D). These data indicate that ATRA treatment does not act on EPO production through cell proliferation.

We then examined the mRNA expressions of several hepatic lineage markers including *delta like non-canonical Notch ligand 1* (*DLK1*), *alpha fetoprotein* (*AFP*), *albumin* (*ALB*), *hepatocyte nuclear factor 4 alpha* (*HNF4A*), *spalt like transcription factor 4* (*SALL4*), *HNF1 homeobox B* (*HNF1B*), *GATA binding protein 4* (*GATA4*) and *Cytokeratin 19* (*CK19*) to evaluate the effects of ATRA on the differentiation status (Fig. 2E). The expression levels of all these markers except *ALB* were not significantly changed by ATRA treatment under hypoxic conditions. Although the expression of *ALB* mRNA was significantly decreased by ATRA treatment under hypoxic conditions, the change was not assumed to have a significant effect on EPO production because of the low frequency of ALB (+) cells (3.3 ± 0.48%, n = 3) compared with AFP (+) cells (98.3 ± 0.25%, n = 3; supplementary Fig. S2A,B). These results imply that changes in the differentiation status had minimal effect on the increase in EPO production caused by ATRA treatment.

Next, considering the possibility of mutual transcription regulation between RA and HIF signals, we examined the mRNA expressions of *HIF1α, HIF2α, PHD1, PHD2, PHD3, RARa* and *RXRa* in hiPSC-EPO cells (supplementary Fig. S2C). Although the mRNA expression levels of *HIF2α, PHD2* and *PHD3* in hiPSC-EPO cells were significantly higher under hypoxic conditions than normoxic conditions, they were not affected by ATRA treatment under hypoxic conditions. Based on these observations, we concluded that the ATRA effect on EPO production does not occur by regulating the transcription of HIFs or PHDs in hiPSC-EPO cells.

### Changes in chromatin structures may be involved in the regulation of EPO expression by RA in hiPSC-EPO cells

**Figure 3:**
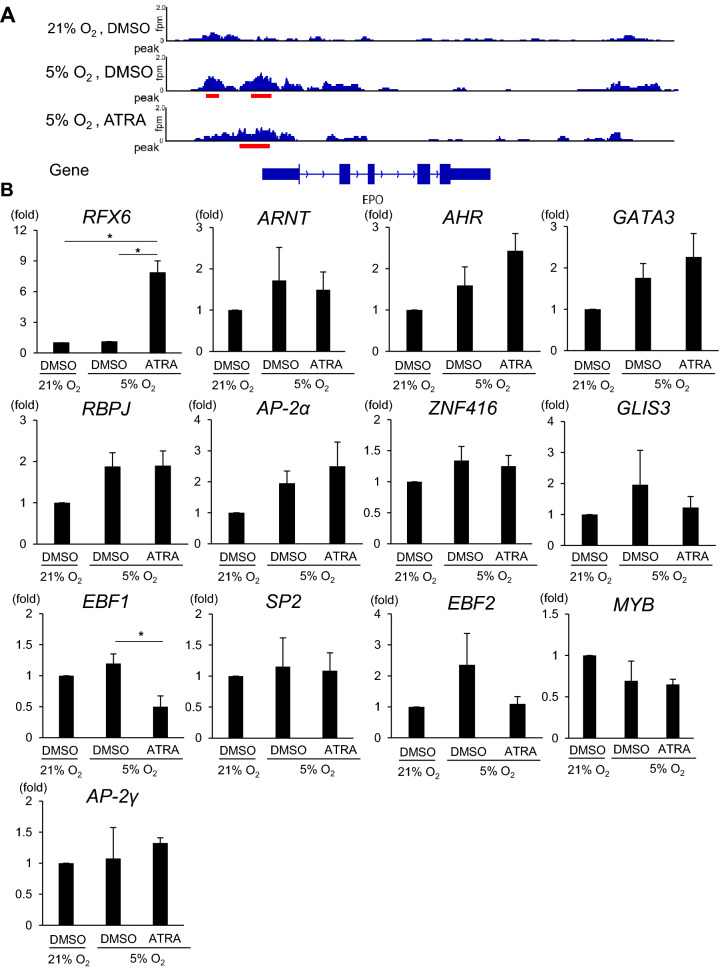
Chromatin structural changes near the EPO promoter in hiPSC-EPO cells. (**A**) Effects of hypoxic signals and ATRA treatment on chromatin formation in hiPSC-EPO cells evaluated by Assay for Transposase-Accessible Chromatin Sequencing (ATAC-seq). The ATAC-seq signals were visualized around the EPO TSS under normoxic (21% oxygen) or hypoxic conditions (5% oxygen) with DMSO treatment or under hypoxic condition with ATRA treatment. The y-axes of the ATAC-seq signals represent fragments per million mapped reads (fpm). Red lines indicate peak call regions. (**B**) qRT-PCR analysis of the mRNA expressions of the genes shown in Table 1. Each value was normalized to the samples treated with DMSO under normoxic conditions. The data from three independent experiments (n = 3) are represented as the means ± SEM. *p < 0.05 by one-way ANOVA with Bonferroni’s test.

Next, we hypothesized that hypoxic signals including HIFs operate epigenetic changes in chromatin, thus allowing RA signals and other unknown transcriptional factors to regulate EPO transcription in hiPSC-EPO cells. Assay for transposase-accessible chromatin sequencing (ATAC-seq) showed that the chromatin region near the EPO transcription start site (TSS) became open after treating hiPSC-EPO cells with DMSO or ATRA under hypoxic conditions (Fig. 3A). Furthermore, a motif analysis showed that the binding sites of 13 genes encoding DNA-binding proteins were identified within the open chromatin region near the EPO TSS under hypoxic conditions, suggesting that these gene sets are putative candidates for the regulation of EPO transcription under hypoxic conditions (Table 1). We thus examined the mRNA expressions of these 13 genes (Fig. 3B). We found the mRNA expressions of *RFX6* and *EBF1* were significantly upregulated and downregulated, respectively, by ATRA treatment under hypoxic conditions. ARNT is known to form a heterodimer with HIFα subunits in the nucleus to promote EPO gene expression. Although both AHR and GATA3 were reported to suppressively regulate EPO production in human hepatoma cell lines^24–26^, the other eleven proteins might be novel EPO transcriptional regulators.

**Table 1:** Candidates genes that may regulate EPO transcription under hypoxic conditions.

Motif	Reference	Consensus
Rfx6(HTH)	Min6b1-Rfx6.HA-ChIP-Seq(GSE62844R)	TGTTKCCTAGCAACM
Arnt:Ahr(bHLH)	MCF7-Arnt-ChIP-Seq(Lo_et_al.)	TBGCACGCAA
GATA3(Zf)	iTreg-Gata3-ChIP-Seq(GSE20898)	AGATAASR
Rbpj1	Panc1-Rbpj1-ChIP-Seq(GSE47459)	HTTTCCCASG
AP-2alpha(AP2)	Hela-AP2alpha-ChIP-Seq(GSE31477)	ATGCCCTGAGGC
ZNF416(Zf)	HEK293-ZNF416.GFP-ChIP-Seq(GSE58341)	WDNCTGGGCA
GLIS3(Zf)	Thyroid-Glis3.GFP-ChIP-Seq(GSE103297)	CTCCCTGGGAGGCCN
EBF1(EBF)	Near-E2A-ChIP-Seq(GSE21512)	GTCCCCAGGGGA
Sp2(Zf)	HEK293-Sp2.eGFP-ChIP-Seq(Encode)	YGGCCCCGCCCC
EBF2(EBF)	BrownAdipose-EBF2-ChIP-Seq(GSE97114)	NABTCCCWDGGGAVH
MYB(HTH)	ERMYB-Myb-ChIPSeq(GSE22095)	GGCVGTTR
AP-2gamma(AP2)	MCF7-TFAP2C-ChIP-Seq(GSE21234)	SCCTSAGGSCAW

### A PHD inhibitor ameliorates renal anemia under the presence of RA in mice

**Figure 4:**
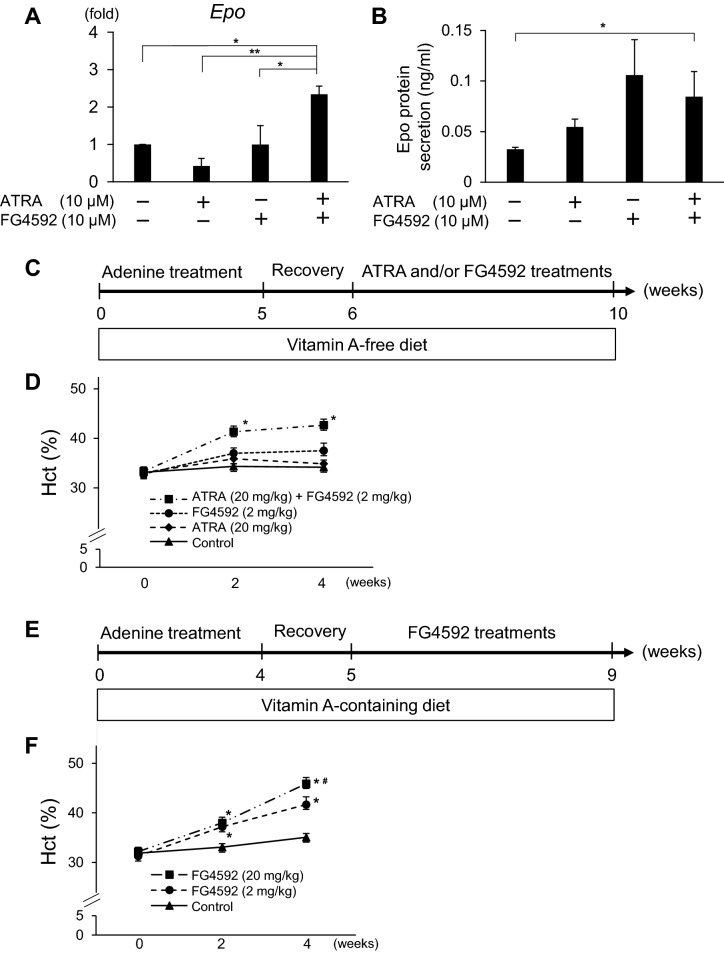
Therapeutic effects of combination treatment with ATRA and FG4592 on renal anemia in adenine-induced mouse models. (**A**,**B**) Effects of ATRA and FG4592 on EPO mRNA expression (**A**) and protein secretion (**B**) by cultured kidney tissues of adult mice under hypoxic conditions (5% oxygen) (n = 4 for mRNA expression and n = 3 for protein secretion). (**C**) Schematic representation of the experimental design for treatment with ATRA and/or FG4592 in renal anemia mice fed a vitamin A-free diet. (**D**) Time-course analysis of the hematocrit (Hct) values in renal anemia mice fed a vitamin A-free diet (n = 5–7). (**E**) Schematic representation of the experimental design for the FG4592 treatment in renal anemia mice fed a vitamin A-containing diet. (**F**) Time-course analysis of the Hct values in renal anemia mice fed a vitamin A-containing diet (n = 5–6). The data are represented as the means ± SEM. *p < 0.05, **p < 0.01 by one-way ANOVA with Bonferroni’s test in (**A**,**B**), *p < 0.01 versus control group in (**D**,**F**) and ^#^p < 0.05 versus the mouse group treated with 2 mg/kg FG4592 by two-way ANOVA with Bonferroni’s test in (**F**).

Finally, we examined whether the additive effects between RA and HIF signals on EPO production are also found *in viv*o*.* We first performed ex vivo experiments using kidney and liver tissues of adult mice. Thinly sliced kidney and liver tissues of three-month-old male mice were cultured on a transwell insert and treated with 10 μM ATRA and 10 μM FG4592 for 24 h. In the cultured adult kidney tissues, combination treatment with ATRA and FG4592 significantly increased *Epo* mRNA expression, whereas monotherapies with ATRA or FG4592 did not (Fig. 4A). Although EPO protein secretion was comparable between FG4592 monotherapy and combination therapy, only combination therapy significantly increased EPO protein secretion compared to control (Fig. 4B), indicating that ATRA potentiated the effects of FG4592 on EPO production. On the other hand, EPO protein secretion was undetectable by any treatment in cultured adult liver tissues (data not shown). These data suggest that the HIF pathway regulates EPO production in cooperation with RA in adult kidney tissues.

Next, we examined the effects of combination treatment with ATRA (20 mg/kg) and FG4592 (2 mg/kg) on adenine-induced renal anemia in mice (Fig. 4C)^27^. After 5 weeks of adenine treatment and a subsequent 1-week recovery period, the mice were treated with only ATRA, FG4592, or the combination of for 4 weeks. In order to strictly evaluate the effects of ATRA on EPO production in vivo, the mice were fed a vitamin A-free diet throughout the experiments. Although body weight was decreased in all mouse groups after 5 weeks of adenine treatment, it recovered by the end of the experiments (data not shown). Consistent with the results of the ex vivo experiments using kidney tissues of adult mice (Fig. 4A,B), the effect of FG4592 was potentiated by ATRA, and only the combination therapy significantly improved renal anemia (Fig. 4D). We also evaluated the effects of FG4592 monotherapy at two different concentrations (2 and 20 mg/kg) in renal anemia mice fed a vitamin A-containing normal diet (Fig. 4E). After 4 weeks of adenine treatment and a subsequent 1-week recovery period, the mice were treated with FG4592. Body weight recovered by the end of the experiments in all mouse groups like in the experiments using the vitamin A-free diet (date not shown). As expected, the FG4592 treatment dose-dependently improved renal anemia in mice (Fig. 4F). These results suggest that HIF signals regulate EPO production under the presence of RA in vivo.

## Discussion

In order to solve the problems associated with renal anemia caused by insufficient EPO production by the kidneys, elucidation of the mechanism underlying the EPO production is needed. Although PHD inhibitors have been developed as a novel therapeutic agent for renal anemia, only regulatory signals and factors from the HIF-PHD pathway for EPO production have been clarified. Makita et al. previously reported that EPO production is regulated by RA signaling in the early stage of fetal liver in mice^14^. Okano et al*.* showed that a human hepatoma cell line, HepG2, increases EPO protein secretion by RA signals, and that the effects were potentiated under hypoxic conditions^28^. However, the association between RA and HIF signals in renal anemia animal models has not been reported. Moreover, cooperative effects between RA signals and PHD inhibitors on EPO production in vitro and in vivo are unknown. Because a sufficient supply of human EPO-producing cells is unavailable due to technical limitations, human hepatoma cell lines that produce EPO in a hypoxia-inducible manner have been used to investigate the mechanisms of EPO production in vitro^29^. However, the EPO production mechanisms of the hiPSC-EPO cells used in this study are expected to be more physiologically relevant than those of tumor cell lines. With this cell model, we show that RA signals regulate the EPO production by hiPSC-EPO cells under hypoxic conditions, an effect enhanced by PHD inhibitors. We confirmed that the effects of a PHD inhibitor on EPO production are potentiated by RA in the cultured kidney tissues of adult mice and that the PHD inhibitor efficiently improves renal anemia in the presence of RA in mice. Vitamin A is a fat-soluble vitamin absorbed through the small intestine in the form of retinol and stored in stellate cells in the liver. Vitamin A deficiency is typically caused by insufficient intake and malabsorption due to chronic diarrhea, bowel resection, pancreatic disorders and cholestatic liver cirrhosis^30–33^. Because RA is a metabolite of vitamin A, our results suggest that vitamin A deficiency should be considered a possible cause of the resistance to PHD inhibitor treatments in renal anemia.

Unlike ATRA, bexarotene did not show additive effects on EPO production with PHD inhibitor treatment in hiPSC-EPO cells, even though both compounds increased EPO production under hypoxic conditions. The RAR/RXR heterodimer is considered to play a crucial role in EPO transcription by binding to direct repeat 2 element in the EPO 3′ enhancer, in which HIF also binds to hypoxia response element (HRE)^14,34^. A previous report showed that RAR and RXR in the heterodimer can regulate the transcription independently and differentially depending on RAREs^35^. Therefore, a possible explanation for the discrepancy between ATRA and bexarotene in the additive effect with PHD inhibitors could be the presence of RAREs regulating EPO transcription, where RXR might need not only HIF-PHD signals but also the recruitment of other transcriptional activators or epigenome changes induced under hypoxic conditions for the additive effect.

Since our results indicate that the additive effects of RA on EPO production with hypoxic signals are not attributable to the proliferation or change in the differentiation status of hiPSC-EPO cells or expression of HIFs and their regulators, we considered the effect of epigenetic changes on EPO expression. It was reported that HIFα subunits bind to HRE in the EPO 3′ enhancer region under hypoxic conditions to recruit the coactivators p300 and CREB-binding protein (CBP), which possess histone acetyltransferase (HAT) activity^36–39^. These coactivators bring about histone modifications besides acting as transcriptional activators. We therefore assumed that predictable chromatin remodeling induced by hypoxic signals enables RA to regulate the transcription of *EPO* mRNA in hiPSC-EPO cells.

The ATAC-seq analysis performed in this study showed that the chromatin region near the EPO TSS becomes accessible in hiPSC-EPO cells stimulated by hypoxic signals. Based on our data, there are three possible mechanisms by which RA regulates the EPO transcription in this condition. First, RA can regulate EPO transcription through the candidate EPO transcriptional regulators that were extracted by our ATAC-seq and motif analyses. Indeed, RA upregulated the mRNA expression levels of *RFX6* under hypoxia and might help candidates including *RFX6* to bind to the region near the EPO TSS that became open under hypoxic conditions. Second, although we have not compared the chromatin states in the presence and absence of RA, RA might regulate EPO transcription by some epigenetic changes in hiPSC-EPO cells. Indeed, it was reported that RARs can regulate epigenetic changes, such as histone methylation or acetylation, by recruiting p300 and other transcriptional co-activators to RAREs^40,41^. Finally, the motif analysis performed in this study identified a RARE near the EPO promotor region (date not shown). However, we did not further examine this region, because the ATAC-seq data showed that the chromatin state in that region was not changed under hypoxia with DMSO or ATRA treatment compared with normoxia. Although this finding makes it less likely that RA acts on the RARE through RAR, we still assumed that RA regulates EPO transcription directly through some RAREs in hiPSC-EPO cells, such as that in EPO 3′ enhancer region, which was previously reported^13^. Further studies should elucidate the detailed molecular mechanisms of EPO transcriptional regulation by RA signals in hiPSC-EPO cells under hypoxic conditions.

There are several limitations in the current study. First, we did not detect an increase in EPO expression by the kidneys or liver or an increase in serum EPO protein concentrations in renal anemia mice (data not shown). The biological half-life of ATRA and FG4592 or changes in the EPO expression or serum EPO concentrations after the administration of these chemicals should be closely examined to identify the best time for the sample collection. Second, since some recent clinical reports suggested that EPO is also produced by the adult liver of CKD patients in compensation for the renal functional loss, as evidenced by the presence of the liver-specific glycosylation pattern in circulating EPO^7,42^, the liver can be a possible target organ in the development of novel therapeutic agents for renal anemia. Our hiPSC-EPO cells are available to elucidate the mechanisms of EPO production especially by the liver. However, there may be some differences in the mechanisms of EPO production between the liver and kidneys^29,43^, which were not addressed in this study. Because our hiPSC-EPO cells are likely to model EPO regulation in the liver, but not the kidney, future studies should develop kidney-lineage EPO-producing cells from hiPSCs and compare the mechanisms of EPO production between the two organs. Third, we have not examined the effects of the 13 factors extracted by ATAC-seq (Table 1) on EPO transcription. Assays including gene knockdown of the 13 factors should be considered. Finally, although some previous reports showed that HNF4A regulates hepatic EPO production by binding to direct repeat 2 element in the EPO 3′ enhancer^10,36^, the detailed roles of HNF4A in hiPSC-EPO cells remain unknown.

In conclusion, we demonstrated that RA potentiates the EPO production induced by HIF signals in hiPSC-EPO cells. The cooperative action between RA and HIF signals was also confirmed in kidney tissues of adult mice ex vivo, and the effects of a PHD inhibitor on renal anemia was augmented by RA in CKD model mice. Thus, our findings using hiPSC-EPO cells and CKD model mice should contribute to elucidating the mechanisms of EPO production and developing efficient therapeutic strategies for renal anemia.

## Materials and methods

### Ethics statement

Experiments using hiPSCs were approved by the ethics committee of the Department of Medicine and Graduate School of Medicine, Kyoto University. Informed consent was obtained from the donor from whom hiPSCs were derived according to the guidelines of the Declaration of Helsinki. All methods were performed in accordance with the institutional guidelines. All experimental protocols involving animals were approved by the CiRA Animal Experiment Committee at Kyoto University and the Animal Experimentation Ethics Committee at Kagawa University. All animal experiments were performed in accordance with the ARRIVE guidelines and the guidelines for the care and use of animals established by Kyoto University and Kagawa University.

### Cell culture

An hiPSC line, 585A1^44^, was maintained under feeder-free conditions with Essential 8 medium (Thermo Fisher Scientific) according to the manufacturer's instructions. For routine passaging, hiPSC colonies were dissociated by an enzymatic method with 0.5 mM ethylenediaminetetraacetic acid (EDTA; Wako). hiPSCs were routinely monitored for mycoplasma contamination.

### In vitro differentiation into EPO-producing cells

The differentiation of hiPSC-EPO cells was performed by modifying our previous protocol^8^. Briefly, hiPSC colonies were dissociated into single cells via gentle pipetting after treatment with 0.5 mM EDTA. Single cells were seeded on Matrigel-coated plates (BD Bioscience) at a density of 4.5 × 10^5^ cells/cm^2^ with Stage 1 medium containing RPMI 1640 (Nacalai Tesque) supplemented with penicillin/streptomycin (500 U/ml; Thermo Fisher Scientific), B27 supplement (2%; Thermo Fisher Scientific), recombinant human/mouse/rat activin A (100 ng/ml; R&D Systems) and CHIR99021 (1 μM; StemRD). Y-27632 (10 μM; Wako) was added to Stage 1 medium for the first 24 h. On day 4, the medium was changed to Stage 2 medium containing KnockOut DMEM (Thermo Fisher Scientific) supplemented with penicillin/streptomycin (500 U/ml), 20% knockout serum replacement (Thermo Fisher Scientific), 1% dimethyl sulfoxide (DMSO; Sigma-Aldrich), 1 mM l-glutamine (Thermo Fisher Scientific), 1% nonessential amino acid (Thermo Fisher Scientific) and 0.1 mM β-mercaptoethanol (Thermo Fisher Scientific). In the experiments shown in Figs. 1B–E, 2, 3, supplementary Figs. S1 and S2, which used the chemical compounds listed in supplementary Table S1, the cells were cultured in vitamin A-free medium containing DMEM/F12-glutamax (Thermo Fisher Scientific) with B27 supplement minus vitamin A (2%; Thermo Fisher Scientific) for 24 h before administration. hiPSC-EPO cells on Stage 2 day 8 were used for the in vitro experiments.

### RT-PCR and real-time quantitative RT-PCR (qRT-PCR)

Both RT-PCR and qRT-PCR were performed as previously reported^45^. Briefly, total RNA was extracted using the RNeasy Kit (Qiagen) according to the manufacturer’s instruction and quantified by Nanodrop 8000 (Thermo Fisher Scientific). cDNA was obtained by reverse transcription using ReverTra Ace (Toyobo). PCR was performed with the Ex-Taq PCR Kit (Takara Bio) according to the manufacturer’s protocol using a thermal cycler (Veriti96-Well Thermal Cycler; Thermo Fisher Scientific). The PCR cycles were as follows. For *β-ACTIN* and *GAPDH*, initial denaturation at 94 °C for 2.5 min, followed by 25 cycles of 94 °C for 30 s, 60 °C for 30 s, 72 °C for 30 s, and a final extension at 72 °C for 10 min. For the other genes, the cycles consisted of an initial denaturation at 94 °C for 2.5 min, followed by 35–40 cycles of 94 °C for 30 s, 60 °C for 30 s, 72 °C for 30 s, and a final extension at 72 °C for 10 min. qRT-PCR was performed using SYBR Green PCR Master Mix (Takara Bio) and the StepOnePlus Real-Time PCR System (Thermo Fisher Scientific). Denaturation was performed at 95 °C for 30 s followed by 40 cycles at 95 °C for 5 s and at 60 °C for 30 s. The threshold cycle method was used to analyze the data for gene expression levels, and the values were calibrated to those of the housekeeping gene *β-ACTIN* or *GAPDH*. The primer sequences used in this study are listed in supplementary Table S2.

### ATAC-seq

DNA was prepared using the Nextera DNA Sample Prep Kit (Illumina) as previously reported^45^. Briefly, after lysing the cells, the nuclear pellet was resuspended into the transposition reaction mix including Tn5 transposase and incubated for 30 min at 37 °C. After that, the transposed DNA fragments were amplified by PCR using barcoded primers. The resulting libraries were then paired-end sequenced (75 bp × 2) on NextSeq 500 (Illumina). After trimming of the adaptor sequences and low-quality bases at the 3′ ends with cutadapt-1.12^46^, the sequenced reads were mapped to the human reference genome (hg38) using Bowtie2 version 2.2.5^47^. Uniquely and properly paired reads were used for further analysis. Duplicated reads and reads falling within blacklisted regions were removed using picard tool version 1.134 (http://broadinstitute.github.io/picard/) and bedtools v. 2.26.0^48^, respectively. The mapped data were scaled to fragment per million mapped reads (fpm) using the genomeCoverageBed command in BEDTools v. 2.26.0 and visualized with Integrative Genomics Viewer (IGV)^49^. Peak calling was performed using MACS2 (2.1.1.20160309)^50^ with default parameters. Motif analysis was performed with homer v4.10.3^51^.

### Measurement of EPO protein secretion

For the quantitative measurement of EPO protein in the culture medium, enzyme-linked immunosorbent assay (ELISA) was performed according to the manufacturer's protocol (ALPCO). Briefly, the culture medium for hiPSC-EPO cells was added into the plate with biotinylated and peroxidase-labeled anti-EPO antibodies. After incubation for 2 h at room temperature, tetramethylbenzidine substrate was added, and the reaction was stopped with 0.5 mM sulfuric acid. The absorbance of the solution was read at 450 nm with a microplate reader (2104 EnVision; PerkinElmer), and the concentration of EPO protein was calculated using the standard curve of lyophilized synthetic EPO protein.

### Immunostaining

Immunostaining of the cultured cells was carried out as previously described^8^. Briefly, cells were fixed with 4% paraformaldehyde/PBS for 20 min at 4℃ and blocked with 1% normal donkey serum (Sigma-Aldrich) and 3% bovine serum albumin (Nacalai Tesque)/PBST (PBS/0.25% Triton X-100) for 30 min at room temperature. The following primary antibodies were incubated overnight at 4℃: Ki67 (BD Biosciences), AFP (Sigma) and ALB (BETHYL). Secondary antibodies (Alexa Fluor 547-conjugated donkey anti mouse IgG; Thermo Fisher Scientific) were incubated for 1 h at room temperature. Fluorescence microscopy (BZ-9000; Keyence) was used to evaluate the stained cells.

### Ex vivo tissue cultures of kidney and liver

Thinly sliced kidney and liver tissues of three-month-old male C57BL/6 mice were cultured on a transwell insert with 0.4 μm polyester membrane (Corning Incorporated) in DMEM/F12 glutamax medium (Thermo Fisher Scientific) supplemented with 10% fetal bovine serum treated with charcoal/dextran (HyClone Laboratories) and penicillin/streptomycin (500 U/ml) under 5% oxygen. The tissues were cultured for 24 h with basal medium before the treatment of each medication.

### Renal anemia model mice

Six-week-old male C57BL/6 mice (CLEA Japan) were maintained in specific pathogen-free facilities under controlled conditions of temperature (24 ± 2 °C) and humidity (55 ± 5%) with a 12-h light–dark cycle. The mice were given a vitamin A-free diet or vitamin A-containing diet and had access to water ad libitum throughout the experiments. The adenine-induced renal anemia models were developed by daily oral gavage of adenine (50 mg/kg body weight in 0.5% methylcellulose) over 5 and 4 weeks for the vitamin A-free and -containing diet mouse groups, respectively. Hematocrit (Hct) levels were measured to confirm renal anemia. After a 1-week recovery period, treatments for renal anemia were performed every day for 4 weeks. First, ATRA (Sigma-Aldrich) and/or FG4592 (Cayman Chemical) treatments were performed on the vitamin A-free diet mouse group. Four mouse groups were prepared: group 1, no treatment (control; n = 6); group 2, ATRA treatment (20 mg/kg; n = 6); group 3, FG4592 treatment (2 mg/kg; n = 6); and group 4, combination treatment with ATRA (20 mg/kg) and FG4592 (2 mg/kg; n = 6). Second, monotherapies with FG4592 were performed on the vitamin A-containing diet mouse group. Three mouse groups were prepared: group 1, no treatment (control; n = 6); group 2, low-dose FG4592 treatment (2 mg/kg; n = 6); and group 3, high-dose FG4592 treatment (20 mg/kg; n = 6). After 2 and 4 weeks of treatment, the Hct levels were measured.

### Statistical analysis

Results are expressed as the mean ± SEM. Multiple-group comparisons were conducted using a one-way or two-way analyses of variance (ANOVA) followed by Bonferroni’s test or Dunnett’s test. Student’s *t*-tests were performed to compare the mean values when the experimental design was comprised of two individual groups. A p value of < 0.05 was considered statistically significant.

## Supplementary information

Supplementary information.
