# Supplementary material for: Retinoic acid regulates erythropoietin production cooperatively with hypoxia-inducible factors in human iPSC-derived erythropoietin-producing cells
Source: Sci Rep. 2021 Feb 16;11:3936. doi: 10.1038/s41598-021-83431-6 (PMC7887226; doi:10.1038/s41598-021-83431-6)
Supplement: Supplementary file 1 — Supplementary information. [file 41598_2021_83431_MOESM1_ESM.pdf]

Supplementary Information

Retinoic acid regulates erythropoietin production cooperatively with hypoxia-inducible factors in human iPSC-derived erythropoietin-producing cells

Naoko Katagiri, Hirofumi Hitomi, Shin-Ichi Mae, Maki Kotaka, Li Lei, Takuya Yamamoto, Akira Nishiyama, Kenji Osafune.

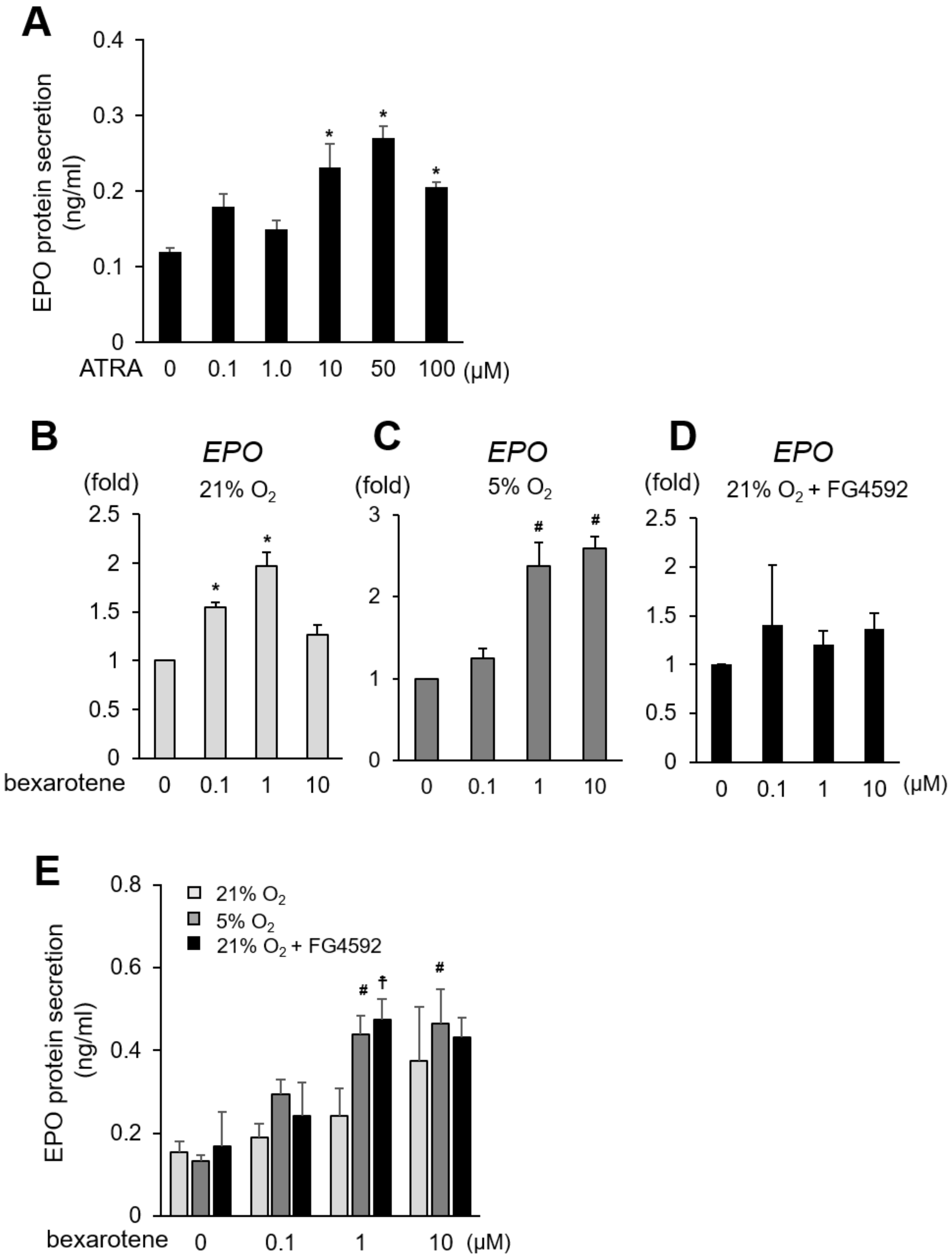

A
EPO protein secretion (ng/ml)
0.4
0.3
0.2
0.1
0
ATRA 0 0.1 1.0 10 50 100 (μM)
B
EPO
(fold)
21% O2
2.5
2
1.5
1
0.5
0
bexarotene 0 0.1 1 10
C
EPO
(fold)
5% O2
3
2
1
0
0 0.1 1 10
D
EPO
(fold)
21% O2 + FG4592
2.5
2
1.5
1
0.5
0
0 0.1 1 10 (μM)
E
EPO protein secretion (ng/ml)
21% O2
5% O2
21% O2 + FG4592
0.8
0.6
0.4
0.2
0
bexarotene 0 0.1 1 10 (μM)

Supplementary Fig. 1. Effects of RA and hypoxic signals on EPO production by hiPSC-EPO cells.

(**A**) Effects of various concentrations of ATRA on EPO protein secretion by hiPSC-EPO cells under normoxic conditions (21% oxygen). (**B** - **E**) Effects of bexarotene treatment on EPO mRNA expression (**B - D**) and protein secretion (**E**) by hiPSC-EPO cells under normoxia (21% oxygen; **B**, **E**, light gray), hypoxia (5% oxygen; **C**, **E**, dark gray), and normoxia with PHD inhibitor treatment (10 μM FG4592: **D**, **E**, black), as analyzed by qRT-PCR and ELISA, respectively. Note that the qRT-PCR analysis in (**B** - **D**) were performed independently. The data from four independent experiments (n=5 for **A** and n=4 for **E**) or three independent experiments (n=3 for **B** - **D**) are represented as the means ± SEM in (**A** - **E**). Statistical analysis was performed using one-way ANOVA with Dunnett's test. *p<0.05 versus the samples treated with DMSO under normoxic conditions in (**A, B**). #p<0.05 versus the samples treated with DMSO under hypoxic conditions in (**C, E**). †p<0.05 versus the samples treated with FG4592 but without bexarotene under normoxic conditions in (**E**).

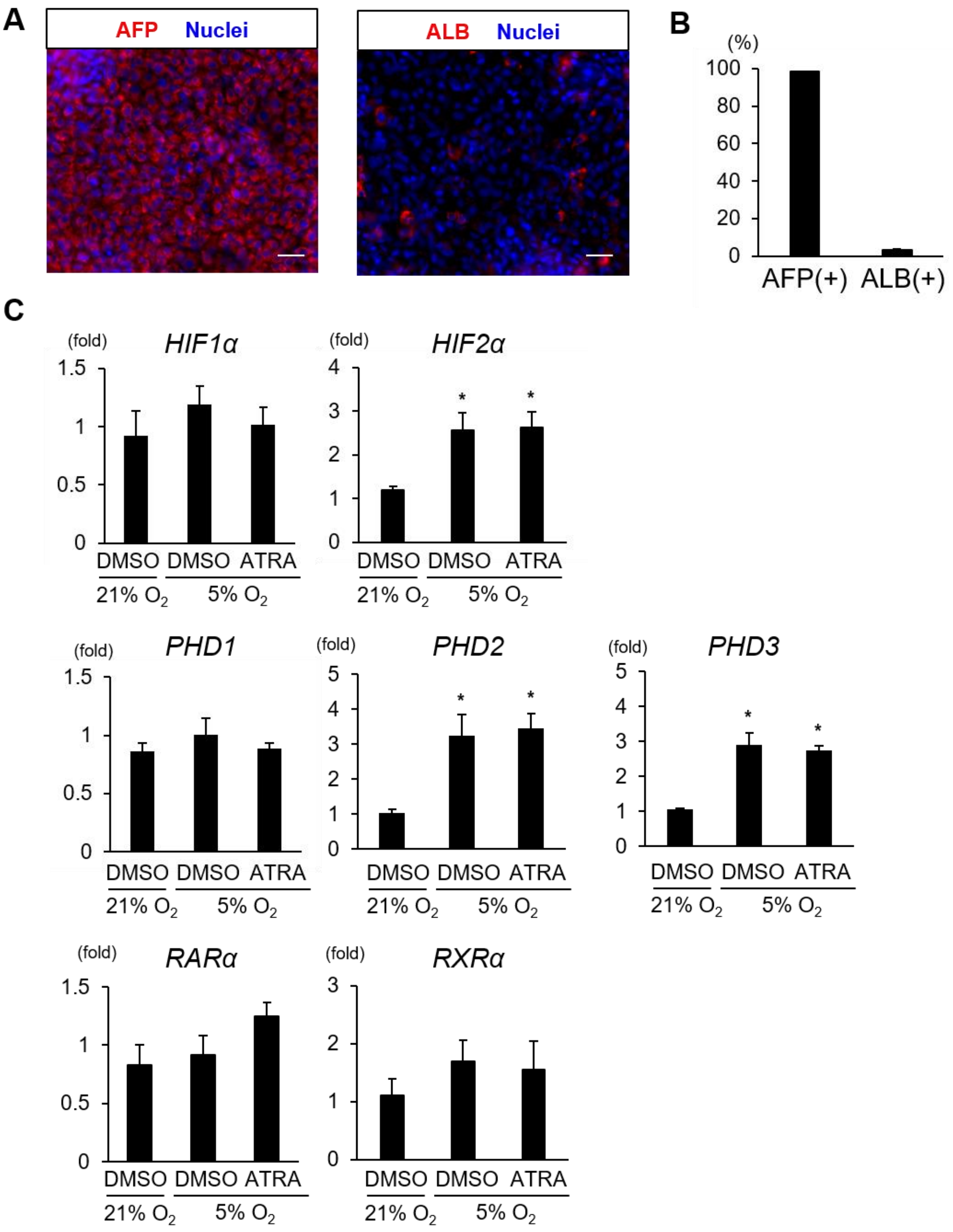

Supplementary Fig. 2. Effects of RA and HIF signals on EPO production by hiPSC-EPO cells.

(**A**) Immunostaining analysis of hiPSC-EPO cells under normoxic conditions (21% oxygen) for AFP and ALB. Scale bars, 50 μm. (**B**) Percentage of AFP(+) or ALB(+) cells in (**A**). (**C**) qRT-PCR analysis of the expressions of *HIF1α*, *HIF2α*, *PHD1*, *PHD2*, *PHD3*, *RARα* and *RXRα* by hiPSC-EPO cells treated with DMSO under normoxic or hypoxic conditions (5% oxygen) or with ATRA under hypoxic conditions. The data from three independent experiments (n=4 for **B** and n=3 for **C**) are represented as the means ± SEM in (**B**, **C**). *$p<0.05$ versus the samples treated with DMSO under normoxic conditions by one-way ANOVA with Bonferroni's test.

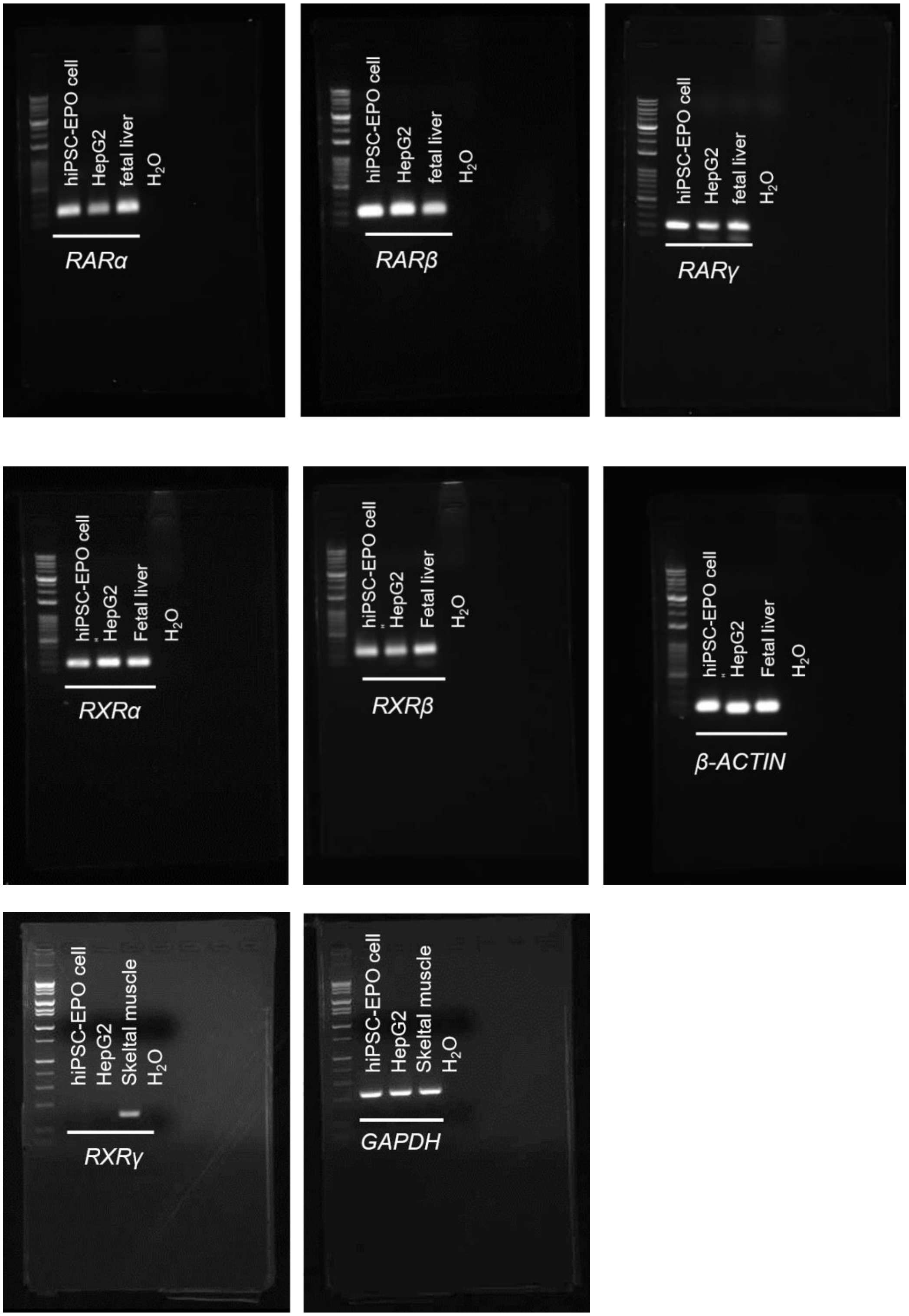

Supplementary Figure 3. Full unedited gel for Figure 1A.

Supplementary Table 1. The chemical compounds used in this study.

**Chemical compound**	**Function**	**Company**
all-trans-retinoic acid (ATRA)	RAR agonist	Sigma-Aldrich
Bexarotene	RXR agonist	R&D
AGN193109	RAR antagonist	SANTA CRUZ
FG4592 (roxadustat)	PHD inhibitor	Cayman Chemical
Molidustat	PHD inhibitor	Cayman Chemical
Daprodustat	PHD inhibitor	Selleck Biotech
deferoxamine (DFO)	PHD inhibitor	Sigma-Aldrich
dimethyloxalylglycine (DMOG)	PHD inhibitor	Sigma-Aldrich

Supplementary Table 2. The primer sequences used in this study.

Gene	Orientation	Primer sequence (5'-3')
EPO	Sense	TGTGGATAAAGCCGTCAGTG
	Antisense	GATTGTTCGGAGTGGAGCAG
Epo	Sense	TTGTGCAGAAGGTCCCAGAC
	Antisense	GCAGTGAAGTGAGGCTACGT
β-ACTIN	Sense	CAATGTGGCCGAGGACTTTG
	Antisense	CATTCTCCTTAGAGAGAAGTGG
GAPDH	Sense	GAAGGTGAAGGTCGGAGTC
	Antisense	GAAGATGGTGATGGGATTTC
Gapdh	Sense	AACTTTGGCATTGTGGAAGG
	Antisense	GGATGCAGGGATGAGTTCT
RARα	Sense	ATGTCCAAGGAGTCTGTGAG
	Antisense	TGTCCCAGAGGTCAATGTC
RARβ	Sense	GGTTTCACTGGCTTGACCAT
	Antisense	GGCAAAGGTGAACACAAGGT
RARγ	Sense	GGCTGTGGGACAAGTTCAGT
	Antisense	GTCGGAGAAGGTCATGGTGT
RXRα	Sense	TTCTCCACCCAGGTGAACTC
	Antisense	GAGCTGATGACCGAGAAAGG
RXRβ	Sense	TCCCACACTTTTCCTCCTTG
	Antisense	ATGGCTCCTACTCCTGCTGA
RXRγ	Sense	TCGTCTGGGCAGATTATTCC
	Antisense	AGGGCTTGATGTCCTCTGAA

DLK1	Sense	TGGCTTCTCAGGCAATTTCT
	Antisense	CTGCAGGTCTTGTCGATGAA
AFP	Sense	AAATGCGTTTCTCGTTGCTT
	Antisense	GCCACAGGCCAATAGTTTGT
ALB	Sense	CGCTATTAGTTCGTTACACCA
	Antisense	TTTACAACATTTGCTGCCCA
HNF4A	Sense	TGCGACTCTCCAAAACCCTC
	Antisense	ATTGCCCATCGTCAACACCT
SALL4	Sense	CAGATCCACGAGCGGACTCA
	Antisense	CCCCGTGTGTCATGTAGTGA
HNF1B	Sense	CCTCTCCTCCAAACAAGCTG
	Antisense	TGTTGCCATGGTGACTGATT
GATA4	Sense	GGAAGCCCAAGAACCTGAAT
	Antisense	GTTGCTGGAGTTGCTGGAA
CK19	Sense	CTCCCGCGACTACAGCCACT
	Antisense	TCAGCTCATCCAGCACCCTG
HIF1α	Sense	CCATTAGAAAGCAGTTCCGC
	Antisense	TGGGTAGGAGATGGAGATGC
HIF2α	Sense	GAAGTCCCGGGATGCTGCGCG
	Antisense	ACTATGTCCTGTTAGCTCCAC
PHD1	Sense	AGCGGGCAGCAGCCAAAGACAAG
	Antisense	TGCCATGCGGCTCTGGGACTG
PHD2	Sense	AGCCCGGCTGCGAAACCATTG
	Antisense	TTCGTCCGGCCATTGATTTTGT

PHD3	Sense	AGATGTGGAGCCCATTTTTG
	Antisense	CAGATTTCAGAGCACGGTCA
RFX6	Sense	TCAGCAGCATTCGTTCACTG
	Antisense	TGGGGTTTGCAAACTGGAAG
ARNT	Sense	CAAGAACCACGGCCTACACT
	Antisense	TAGCTGGCCAGTCCATCTCT
AHR	Sense	AGTCTAATGCACGCCTGCTT
	Antisense	TGGCAGGAAAAGGGTTGGTT
GATA3	Sense	TCCTGTGCGAACTGTCAGAC
	Antisense	CGAGCTGTTCTTGGGGAAGT
RBPJ	Sense	AGTTACTGGCATGGCACTCC
	Antisense	TCCAGGAAGCGCCATCATTT
AP-2α	Sense	AAGAGTTCACCGACCTGCTG
	Antisense	AGGGCCTCGGTGAGATAGTT
ZNF416	Sense	ATGTGTCTTGCAGGAGCTCC
	Antisense	ATCAGCTTCCAGCAGAACCC
GLIS3	Sense	CAACGCCCGCTATAAACTGC
	Antisense	CGGATGCTGGCACAAATACG
EBF1	Sense	CGCAACTCAAGCAGCGTATC
	Antisense	CCAAATTGGACATTGCGGCA
SP2	Sense	GCCCAATCTCACCAACCAGA
	Antisense	CACTGGCGTTCACAAGGTTG
EBF2	Sense	AGGAGACGATTTGCTGGCAA
	Antisense	CCTGACCGAATCCATCTCCG

MYB	Sense	ACAGATGGGCAGAAATCGCA
	Antisense	GCTGGCTGGCTTTTGAAGAC
AP-2γ	Sense	ACCCACACACTTAGCCATTG
	Antisense	AGCGGACACAAAAACCAACC
